# Mitochondrial tRNA mutations in Chinese children with tic disorders

**DOI:** 10.1042/BSR20201856

**Published:** 2020-12-22

**Authors:** Peifang Jiang, Yinjie Ling, Tao Zhu, Xiaoying Luo, Yilin Tao, Feilong Meng, Weixin Cheng, Yanchun Ji

**Affiliations:** 1 Department of Neurology, The Children’s Hospital, Zhejiang University School of Medicine, National Clinical Research Center For Child Health, Hangzhou, China; 2 Department of Pediatrics, First People’s Hospital of Huzhou, Huzhou, China; 3 Department of Critical Care Medicine, Sir Run Run Shaw Hospital, School of Medicine, Zhejiang University, Hangzhou, China; 4 Division of Medical Genetics and Genomics, The Children’s Hospital, Zhejiang University School of Medicine, National Clinical Research Center for Child Health, Zhejiang, China; 5 Institute of Genetics, Zhejiang University, Hangzhou, Zhejiang, China

**Keywords:** Chinese, genetics, molecular biology, pathophysiology, Tourette’s disorder

## Abstract

Aim: To conduct the clinical, genetic, and molecular characterization of 494 Han Chinese subjects with tic disorders (TD).

Methods: In the present study, we performed the mutational analysis of 22 mitochondrial tRNA genes in a large cohort of 494 Han Chinese subjects with TD via Sanger sequencing. These variants were then assessed for their pathogenic potential via phylogenetic, functional, and structural analyses.

Results: A total of 73 tRNA gene variants (49 known and 24 novel) on 22 tRNA genes were identified. Among these, 18 tRNA variants that were absent or present in <1% of 485 Chinese control patient samples were localized to highly conserved nucleotides, or changed the modified nucleotides, and had the potential structural to alter tRNA structure and function. These variants were thus considered to be TD-associated mutations. In total, 25 subjects carried one of these 18 putative TD-associated tRNA variants with the total prevalence of 4.96%.

Limitations: The phenotypic variability and incomplete penetrance of tic disorders in pedigrees carrying these tRNA mutations suggested the involvement of modifier factors, such as nuclear encoded genes associated mitochondrion, mitochondrial haplotypes, epigenetic, and environmental factors.

Conclusion: Our data provide the evidence that mitochondrial tRNA mutations are the important causes of tic disorders among Chinese population. These findings also advance current understanding regarding the clinical relevance of tRNA mutations, and will guide future studies aimed at elucidating the pathophysiology of maternal tic disorders.

## Introduction

Tic disorders (TD) are a form of neuropsychiatric condition wherein individuals suffer from repetitive involuntary motor or vocal tics [1,2]. TD are thought to develop through the interactions between both genetic and environmental variables. Such tics affect up to 25% of children, although individuals of any age can be affected [3,4]. TD rates are significantly higher in boys than in girls. The DSM-V includes three different TDs, including Tourette’s Syndrome (TS), chronic tic disorder (CTD), and provisional tic disorder (PTD) [5]. From a genetic perspective, most researchers agree that TDs have a complex genetic basis, with high degrees of locus and allelic heterogeneity and polygenic inheritance. Most efforts to identify genetic mutations associated with TD risk have centered on nuclear genes which mutations in IMMP2L, CNTNAP2, SLITRK1, NLGN4X, and MRPL3 have been linked to TD risk [6–10]. IMMP2L codes for inner membrane peptidase subunit 2, which is a protease that is present in mitochondria and cleaves sorting signals from proteins within the mitochondrial membrane [6]. MRPL3 encodes for mitochondrial ribosomal protein L3, which is essential for mitochondrial protein synthesis even though it is a nuclear genome [10]. The fact that mutations in both of these mitochondrial genes are closely linked to TD suggests that mitochondrial dysfunction may be a key regulator of TD development [11]. Within the mitochondrial genome, tRNA mutations are common and have the potential to cause structural or functional changes in the mutated tRNA molecules. Such tRNA mutations can in turn lead to altered RNA processing or nucleotide modification, in addition to disrupting normal tRNA metabolism, thereby potentially resulting in mitochondrial dysfunction that can in turn drive TD development [12,13].

While some past studies have conducted relatively focused analyses of particular pedigrees in order to identify mutated nuclear genes encoding mitochondrial proteins, further research is needed to identify TD-related mutations in the 22 different mitochondrial tRNA genes, with a large population size being essential to reliably define such genetic relationships. In addition, how these mutations alter tRNA functionality and how this in turn leads to TD development remains to be fully elucidated. In the present study, we conducted the systematic genetic screening of 494 individuals of Han Chinese ethnicity with known tic disorders who were negative for known pathogenic mutations in nuclear genes were identified. This population was then used to screen for pathogenic mutations in the 22 mitochondrial tRNA genes, leading to the identification of 73 mitochondrial tRNA variants across these 22 tRNA genes. These variants were then assessed for their pathogenic potential via phylogenetic, functional, and structural analyses, and by identifying variants with frequencies of <1% in a population of 485 control individuals from the same region. This analysis led to the identification of 18 potential TD-associated tRNA variants in 25 of the patients included in the present study. We additionally conducted clinical and genetic analyses of the probands carrying these potentially TD-associated mutations.

## Methods

### Participant recruitment

In total, 494 unrelated children that had been diagnosed with TDs and 485 age-, gender-, and region-marched controls from the Children’s hospital, Zhejiang University School of Medicine were recruited for the present study through a program screening for the genetic basis of TDs in Chinese children. Through interviews with participants and guardians, and by conducting physical examinations, participant and family histories of TDs and other clinical conditions were assessed. The present study was conducted in accordance with the Declaration of Helsinki. All participants provided informed consent to participate and to provide blood samples, with the Ethics Committee of the Institutional Review Board of the Children’s Hospital, Zhejiang University School of Medicine having approved the present study. The TDs were evaluated by using the inclusion and exclusion criteria. Inclusion criteria were as follows: The included population must meet all the criteria of DSM-V-TR. We assessed the severity of tics with the Yale Global Tic Severity Scale (YGTSS) (Leckman et al., 1989). The exclusion criteria were as follows: a history of pervasive developmental disorder (PDD), bipolar disorder, major depressive disorder, psychotic disorders, OCD; a history of organic brain disease, seizure disorder, or other neurological disorder; inflammatory diseases or currently using anti-inflammatory medications; an IQ below 70. Control subjects could not meet criteria for any current or past DSM-IV-TR psychiatric disorder, and they showed no other psychiatric disorders and inflammatory diseases.

### Mitochondrial tRNA mutations analyses

After collecting peripheral blood samples from study participants, a Paxgene Blood DNA Isolation Kits (QIAGEN) was used to isolate genomic data. PCR was then used to amplify fragments of all 22 tRNA genes in these patients with appropriate primer pairs, as in previous studies [14]. In individuals found to have potential mutations in these tRNA genes, fragments that spanned the remainder of the mitochondrial genome were also amplified via PCR and were used to estalish the mtDNA haplogroups of these individuals. Bidirectional sequencing in both directions was conducted in order to confirm amplicon sequences, after which sequences were compared with the most recent Cambridge consensus sequence (GenBank accession number: NC_012920). Direct PCR product and subsequently analyzed by direct sequencing in an ABI 3700 automated DNA sequencer (Applied Biosystems; Thermo Fisher Scientific, Inc., Waltham, MA, U.S.A.) using the Big Dye Terminator Cycle Sequencing Reaction Kit (Thermo Fisher Scientific, Inc.).

### Structural analysis

Stem and loop structures were defined based on published human mitochondrial tRNA secondary structures, with tertiary structure interactions for these tRNA molecules being determined by referring to the relevant literature [15,16].

### Phylogenetic analysis

An interspecies analysis was conducted by comparing mitochondrial DNA sequences across 16 different vertebrate species (http://trna.bioinf.uni-leipzig.de/DataOutput/) shown in Supplementary Table S1, with a resultant conservation index (CI) value being defined based on the percentage of species with identical nucleotides to those in humans at a given position.

### Haplogroup analysis

The PhyloTree database was used to assign subjects to mitochondrial haplogroups based on their complete mtDNA sequences [17,18].

## Results

### Clinical features

For the present study, 494 total subjects with TDs (91 female, 403 male) were recruited, with an average age of 7.8 years (range: 6 months – 14 years). The age at which TDs first arose in these patients were ranged from 0 to 5 years (129 subjects), 6 to 11 years (335 individuals), and 12 to 18 years (31 individuals). Participants suffered a range of classical TD symptoms, including 415 participants that experienced involuntary blinking, 246 that were affected by involuntary grimacing, 234 that suffered involuntary throat clearing/phonation, 131 that involuntarily shrugged their shoulders, 140 that suffered from involuntary head shaking, and 26 that exhibited involuntary spitting. Based on the criteria outlined in the DSM-V, 146 patients were diagnosed with TS, 134 with CTD, and 214 with PTD. In addition, we recruited 485 age- and gender-matched control children from this same region. Controls had no personal or family history of TDs, and were an average of 9 years old (range: 2–15).

### Identification of mitochondrial tRNA mutations

Through the sequencing of the 22 mitochondrial tRNA genes in both control and TD subjects, we were able to identify 73 different nucleotide variants in affected patients, including 24 novel variants and 49 known variants. The distribution of these mutations was as follows: 4 variants in MT-TA gene, 9 variants in MT-TC gene, 1 variant in MT-TD gene, 5 variants in MT-TE gene, 2 variants in MT-TF gene, 4 variants in MT-TG gene, 5 variants in MT-TH gene, 1 variant in MT-TK gene, 1 variant in MT-TL1 gene, 2 variants in MT-TL2 gene, 1 variant in MT-TM gene, 3 variants in MT-TP gene, 6 variants in MT-TQ gene, 4 variants in MT-TR gene, 2 variants in MT-TS1 gene, 2 variants in MT-TS2 gene, 14 variants in MT-TT gene, 2 variants in MT-TV gene, 2 variants in MT-TW gene, and 3 variants in MT-TY gene.

### Mitochondrial tRNA variant evaluation

**Figure 1:**
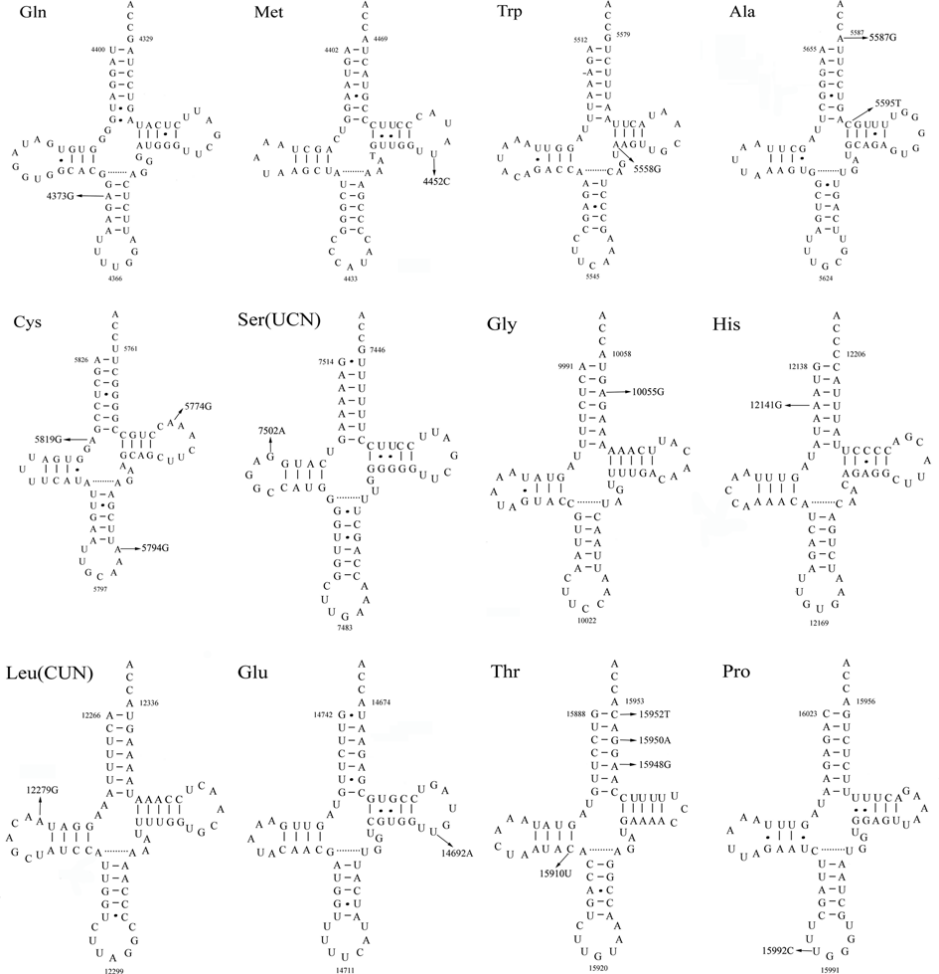
Mitochondrial putative tRNA variants in Han Chinese subjects with TDs Circled numbers represent the nucleotide positions according to the conventional tRNA numbering system. Tertiary interactions between nucleotides are indicated by dotted lines. Arrows indicate the position of the variants in the tRNA.

We next assessed the potential pathogenicity of these tRNA variants with potentially pathogenic variants being those that met the following criteria: (1) the CI value > 75%, consistent with evolutionary conservation at a given locus, as suggested by Ruiz-Pesini and Wallace; (2) variants that were present in <1% of control patients; and (3) these variants were predicted to result in alterations in the structure and/or function of tRNA molecules. We first compared mitochondrial tRNA sequences to matched sequences in 16 vertebrate species to establish the corresponding CI values, which ranged from 12 to 100% for the analyzed loci (Table 1). Of these 73 tRNA variants, 32 had a CI value >75% consistent with their evolutionary conservation. We next assessed the allelic frequencies of these 41 variants in the 485 individual control study population, revealing 18 variants to not be detectable in any control subjects, 12 to be present in <1% of controls, and 2 to be present in >1% of controls. The 18 variants not detectable in control subjects were considered as the most likely pathogenic mutations. The locations of these variants within tRNA secondary structures are shown in Figure 1. Typically, mitochondrial tRNA molecules have a clover-like morphology with acceptor (ACC), anticodon (AC), and T stem domains, in addition to TψC(T), dihydrouridine (D) and anticodon (AC) regions. We found that of these 18 variants, 7 localized to loop regions, while 9 localized to stem regions and 2 localized to terminal or junctional regions. We assessed stem region variants based on predicted base changes according to Watson–Crick (WC) base-pairings. Of these 9 variants (1 in the AC stem, 5 in the ACC stem, 1 in the D stem, and 2 in the T stem), all 9 variants disrupted normal WC base pairing. This analysis thus suggested that these 18 tRNA mutations, which were evolutionarily conserved, were not detected in control patients, and were predicted to disrupt tRNA structure and function, were possible pathogenic mutations. The remaining 55 identified variants were polymorphisms that were either not evolutionarily conserved and/or were present in control patients.

**Table 1:** Summary of Clinical data of 494 tic disorder patients

Variable	Total (*n*=494)	Rate (%)
Gender		
Male	403	81.58
Female	91	18.42
Age (years)		
<6	129	26.11
6–12	334	67.61
12–18	31	6.28
Category		
Tourette’s disorder	146	29.55
Chronic motor or vocal tic disorder	134	27.13
Provisional tic disorder	214	43.32
Symptom		
Blinking	415	84.01
Facial grimacing	246	49.80
Throat clearing/Phonation	234	47.37
Shrug shoulders	131	26.52
Shake head	140	28.34
Spit	26	5.26
Previous medicinal treatment		
0 drug	248	50.20
1 drugs	112	22.67
2 drugs	101	20.45
3 drugs	24	4.86
4 drugs	9	1.82

### Whole mitochondrial genome analysis of the 25 subjects carrying putative tRNA mutations

Of the 494 TD patients in the present study, 25 (5.06%) were found to be carriers of the potential tRNA mutations identified through this analysis (Table 2). We therefore next analyzed these individuals to identify other possible tRNA variants within their mitochondrial genomes. In so doing, we identified 5 variants in the acceptor stem region predicted to disrupt base pairing (m.10055A>G in tRNA^Gly^, and m.12141A>G, m.15948A>G, m.15950G>A, m.15952C>T in tRNA^His^). We further found the m.15910C>T variant in the D-stem of tRNA^Thr^ which was predicted to interfere with base pairing, and the anticodon stem m.4373T>C variant in tRNA^Gln^ that was predicted to interfere with tRNA stability. Furthermore, the T-stem m.5558A>G and m.5595G>A variants were predicted to adversely impact tRNA^Trp^ structure. We also detected 7 loop region variants, including m.7502C>T in tRNA^Ser(UCN)^ and m.12279A>G(A14) in the D-loop region of tRNA^Leu(CUN)^, which were predicted to interfere with cognate aminoacyl-tRNA synthetase-mediated tRNA recognition. The 5′ anticodon m.15992A>G (A34) variant in tRNA^Pro^ was also identified, as was the m.5794T>C variant in tRNA^Cys^. As these latter variants were present at the anticodon end, they may alter codon-recognition and/or tRNA stability. The m.4452T>C and m.14692A>G variants at T-loop conventional position (55) may alter tRNA tertiary structure and impair aminoacylation. We next assigned these 25 patients to Eastern Asian mitochondrial haplogroups according to the PhyloTree database. A total of 305 mtDNA variants were identified in these 25 probands, including 24 variants in 12S and 16S rRNA regions, 81 in control regions, 24 in tRNA genes, 173 in protein-coding genes, and 3 in noncoding regions. These probands were assigned to the A, B, C, D, F, G, M, N, R, and Z haplogroups (van Oven and Kayser, 2009).

**Table 2:** Mitochondrial tRNA variants in a cohort of 494 Chinese Han subjects with tic disorders

Gene	Position	Conservation	Replacement	Watson–Crick base-pairing	No. of tRNA nucleotides	No. of 494 patients (%)	No. of control subjects	Location of structure	Previously reported^*^
Putative mutation									
*MT-TQ*	4373	100	T-C	A-U↓	28	1(0.20)	0	Anticodon stem	N
*MT-TM*	4452	100	T-C		55	1(0.20)	0	T-loop	Y
*MT-TW*	5558	100	A-G	A-U↓	49	2(0.4)	0	T-stem	Y
*MT-TA*	5587	100	T-C		73	2(0.4)	0	ACC terminus	Y
	5595	100	G-A	C-G↓	65	1(0.20)	0	T-stem	N
*MT-TC*	5774	93	T-C		59	1(0.20)	0	T-loop	Y
	5794	86	T-C		38	1(0.20)	0	Anticodon loop	N
	5819	100	T-C		8	1(0.20)	0	D-A junction	N
*MT-TS1*	7502	86	C-T		14	1(0.20)	0	DHU-loop	Y
*MT-TG*	10055	100	A-G	A-U↓	70	2(0.4)	0	ACC-stem	N
*MT-TH*	12141	82	A-G	A-U↓	4	1(0.20)	0	ACC-stem	N
*MT-TL2*	12279	100	A-G		14	2(0.4)	0	DHU-loop	N
*MT-TE*	14692	88	A-G		55	1(0.20)	0	T-loop	N
*MT-TT*	15910	100	C-T	C-G↓	25	1(0.20)	0	DHU-stem	N
	15948	82	A-G	A-U↓	68	1(0.20)	0	ACC-stem	N
	15950	100	G-A	G-C↓	70	1(0.20)	0	ACC-stem	N
	15952	93	C-T	C-G↓	72	2(0.4)	0	ACC-stem	N
*MT-TP*	15992	100	A-G		34	3(0.61)	0	Anticodon loop	N
other mutation									
*MT-TF*	593	29	T-C		17	2(0.4)	2	D-loop	Y
	636	43	A-G		62	1(0.20)	0	T-loop	N
*MT-TV*	1607	59	T-C	C-G↑	6	1(0.20)	0	ACC-stem	N
	1664	29	G-A	A-U↑	67	1(0.20)	1	ACC-stem	Y
*MT-TL1*	3290	32	T-C		59	2(0.4)	2	T-loop	Y
*MT-TQ*	4363	79	T-C		38	1(0.20)	3	AC loop	Y
	4369	59	A-G		32	1(0.20)	0	Anticodon loop	N
	4385	68	A-G		16	1(0.20)	1	D-loop	N
	4386	46	T-C		15	7(1.41)	13	D-loop	Y
	4394	44	C-T		7	5(1.01)	2	ACC-stem	Y
*MT-TW*	5567	65	T-C	U-A↓	61	1(0.20)	0	T-stem	Y
*MT-TA*	5601	59	C-T		59	10(2.02)	8	T-loop	Y
	5628	93	T-C	U-A↓	31	2(0.4)	3	Anticodon stem	Y
*MT-TC*	5773	24	G-A		61	1(0.20)	2	T-loop	Y
	5783	100	G-A	G-C↓	50	2(0.4)	1	T-stem	Y
	5786	50	T-C		46	1(0.20)	0	Variable loop	Y
	5814	85	T-C	A-U↓	13	2(0.4)	1	D-stem	Y
	5821	65	G-A	G-C↓	6	13(2.63)	10	ACC-stem	Y
	5823	29	A-G	G-C↑	4	2(0.4)	0	ACC-stem	Y
*MT-TY*	5836	83	A-G	G-C↑	63	1(0.20)	2	T-stem	Y
	5843	88	A-G		54	1(0.20)	1	T-loop	Y
	5878	64	delT		14	2(0.4)	0	DHU-loop	N
*MT-TS1*	7492	68	C-T	U-A↑	27a	1(0.20)	1	D-A junction	Y
*MT-TD*	7572	29	T-C		60	1(0.20)	2	T-loop	N
*MT-TK*	8343	41	A-G		54	1(0.20)	4	T-loop	Y
*MT-TG*	9992	49	C-T	C-G↓	2	1(0.20)	1	ACC-stem	N
	10007	49	T-C		19	1(0.20)	0	D-loop	Y
	10031	51	T-C		44	7(1.41)	6	Variable loop	Y
*MT-TR*	10410	12	T-C		6	2(0.4)	4	ACC-stem	Y
	10411	41	A-G	A-U↓	7	1(0.20)	0	ACC-stem	Y
	10454	56	T-C		55	3(0.61)	4	T-loop	Y
	10463	94	T-C		67	1(0.20)	1	ACC-stem	Y
*MT-TH*	12153	59	C-T		16	1(0.20)	1	D-loop	Y
	12172	93	A-G		38	3(0.61)	2	AC loop	Y
	12190	79	A-G		57	1(0.20)	1	T-loop	N
	12192	12	G-A		59	3(0.61)	1	T-loop	N
*MT-TS2*	12216	39	C-T		10	1(0.20)	0	D-loop	N
	12231	63	C-T	A-U↑	39	1(0.20)	0	Anticodon stem	N
*MT-TL2*	12280	59	A-G		15	1(0.20)	1	D-loop	Y
*MT-TE*	14687	93	A-G		60	1(0.20)	1	T-loop	Y
	14693	98	A-G		98	10(2.02)	7	T-loop	Y
	14696	93	A-G	G-C↑	51	1(0.20)	1	T-stem	Y
	14727	64	T-C		16	1(0.20)	1	DHU-loop	Y
*MT-TT*	15889	41	T-C	U-A↓	2	2(0.4)	0	ACC-stem	Y
	15900	73	T-C	U-A↓	13	1(0.20)	1	D-stem	Y
	15924	93	A-G	A-U↓	39	9(1.82)	7	ACC stem	Y
	15927	49	G-A	G-C↓	42	8(1.62)	16	ACC stem	Y
	15928	76	G-A	G-C↓	43	2(0.4)	2	ACC stem	Y
	15930	22	G-A		45	4(0.81)	15	Variable loop	Y
	15932	64	T-C		47	3(0.61)	0	T-loop	Y
	15937	50	A-G		53	1(0.20)	0	T-loop	Y
	15940	24	T-C		59	4(0.81)	3	T-loop	Y
	15951	68	A-G	A-U↓	71	1(0.20)	4	ACC-stem	Y
*MT-TP*	15968	27	T-C	U-A↓	61	2(0.4)	2	T-stem	Y
	16000	19	G-T	A-U↑	26	1(0.20)	1	D-A junction	Y

* Y means Yes; N means No.

## Discussion

Herein we analyzed 494 children with TD and thereby identified 73 total variants through analyzing mitochondrial tRNA variants. By focusing only on variants that were evolutionarily conserved, not present in control subjects, and predicted to induce functional or structural changes in tRNA molecules we were ultimately able to identify 18 potentially pathogenic mutations in 12 mitochondrial tRNAs in these patients. Of the 494 affected participants (including TS, CTD, and PTD) in the present study, 25 harbored one of these 18 pathogenic variants, with an overall frequency of 3.64% in this Han Chinese population. These tRNA genes may thus represent a mutational hotspot associated with TD incidence. One mutation noted in the study participants was the m.5587A>G variant in the 3′ end of the tRNA^Ala^ gene, which may impact tRNA structure and thereby alter amino acid translational efficiency, potentially disrupting protein synthesis and thereby leading to disrupted mitochondrial functionality. Mitochondrial respiratory chain proteins may be particularly susceptible to such dysfunction may be the mitochondrial respiratory chain proteins involved in oxidative phosphorylation [19,20], and consequent disruption of oxidative phosphorylation may in turn lead to a series of pathological changes including reactive oxygen production and decreased nitric oxide usage within mitochondria. In one previous report, a 28-year-old female harboring this mutation exhibited a 16-year history of progressive gait instability, dysarthria, hearing loss, muscle cramps, and myalgia [21]. This same mutation has also been tentatively linked to Leber’s hereditary optic neuropathy and hypertension in Chinese pedigrees [20,22].

The T-stem mutations m.5558A>G and m.5595G>A identified in our analyses are also predicted to impact tRNA^Trp^ structure and function, as is the m.4317A>G mutation in tRNA^Ile^ which was the most common variant in this study, having detected in 3 members(0.34%) of the study population [23]. This mutation has previously been described in the context of cardiomyopathy, and localizes to a highly conserved adenine (A59) in the T-loop of tRNA^Ile^, resulting in T-stem reorganization. *In vitro*, this mutation has been shown to interfere with the 3′ end processing of the precursor to tRNA^Ile^, leading to reduced CCA-addition of this tRNA. Indeed, impaired tRNA^Ile^ conformation, stability, and aminoacylation efficiency were detected in lymphoblastoid cell lines. In a Chinese pedigree of individuals suffering from deafness, we found that this m.4317A>G mutation synergized with the m.1555A>G mutation leading to increased penetrance, with cell lines bearing both of these mutations exhibiting more significant mitochondrial dysfunction than those harboring only the m.1555A>G mutation [23]. We additionally identified 5 variants (m.10055A>G in tRNA^Gly^, m.12141A>G, m.15948A>G, m.15950G>A, m.15952C>T in tRNA^His^) in the acceptor stem region that disrupted base pairings, potentially impairing tRNA function in a manner similar to the m.7511T>C and m.12201T>C mutations, caused defects in respiratory capacity in mutant cells. Furthermore, marked decreases in the levels of mitochondrial ATP and membrane potential were observed in mutant cells. Such mitochondrial dysfunction caused an increase in the production of reactive oxygen species in the affected cells. [24,25]. We further identified the m.15910C>T variant in the D-stem of tRNA^Thr^ and determined that this variant altered base pairing in the region, likely disrupting tRNA stability and functionality, as in the case of the m.7505T>C and m.10003T>C mutations [26–28]. The m.4373T>C anticodon stem variant was also predicted to adversely impact tRNA^Gln^ stability in a manner similar to the m.15927G>A mutation in tRNA^Thr^ [29].

Many of the detected variants were present in the D- or T-loop regions, and therefore had the potential to disrupt the tertiary structure in these regions, thus adversely impacting tRNA stability and folding. Of the 7 loop variants, 2 were in the D-loop, 2 were in the AC loop, and 3 were in the T-loop. In addition, 4 variants including tRNA^Ser(UCN)^ 7502C>T and tRNA^Leu(CUN)^ 12279A>G occurred at the highly conserved positon (14), thereby disrupting the tertiary interactions between the D- and T-loops necessary for L-shaped tRNA stability. Furthermore, the m.7502C>T and m.12279A>G variants occurred at the 14A-8U interaction site, which is important for cognate aminoacyl tRNA synthetase recognition. The m.7502C>T and m.12279A>G variants have the potential to disrupt the aminoacylation and steady state stability of tRNA^Ser(UCN)^ and tRNA^Leu(CUN)^, as has been shown for the m.3243A>G mutation [30,31]. Other variants were found within the AC loop, which is a functionally important region. These variants included m.15992A>G (position 34) at the 5′ end of the anticodon of tRNA^Pro^ and m.5794T>C (position 38) at 3′ end of tRNA^Phe^ in highly conserved regions. Methylation and thiolation are common modifications for mitochondrial tRNAs at position 34, and as such variants affecting this site have the potential to interfere with anticodon-codon recognition and tRNA structural/functional stability. The m.14692A>G and m.4452T>C variants can disrupt tRNA tertiary structure and impair aminoacylation, as with the m.8344A>G mutation in tRNA^Lys^ [32,33]. We have recently found that the m.14692A>G mutation leads to destabilized base pairing (18A-Ψ55) and the impairment of mitochondrial translation caused defective respiratory capacity, with marked reductions in the activities of respiratory complexes I and IV. Furthermore, marked decreases in mitochondrial ATP and membrane potential were observed in mutant cells. These mitochondrial dysfunctions thereby enhanced reactive oxygen species in these mutant cells. thereby disrupting tRNA^Glu^ tertiary structure. Reductions in the number of steady-state cells carrying the m.14692A>G can thus contribute to mitochondrial dysfunction [32]. Moreover, the m.5819T>C mutation located in the A-D junction of mitochondrial tRNA^Cys^ may perturb the structure of the ACC-stem and D stem junction similar with the m.7526A>G mutation in tRNA^Asp^,which has been shown to resulted in isolated mitochondrial myopathy [34].

## Conclusions

Both mitochondrial and nuclear mutations can predispose individuals to TDs, as these genetic interact with environmental variables to mediate the eventual development of these disorders. Mutated isoforms of specific nuclear modifier genes have been reported to be important drivers of TD development, and as such we hypothesize that the tRNA mutations identified in the present study do not exhibit complete penetrance. Instead, these mutations in the mtDNA mutations probably require additional input associated with mitochondrial haplotype, nuclear modifier gene activity, and environmental/epigenetic regulation in order to mediate TD development in affected individuals.

## Supplementary Material

Supplementary Table S1

## Data Availability

All data generated or analyzed during this study are included in this published article. The links to the data generated: https://www.ncbi.nlm.nih.gov/bioproject/PRJNA675670.

## Abbreviations

A-D junction: ACC-stem and D stem junction
CI: conservation index
CTD: chronic tic disorder
PTD: provisional tic disorder
TD: Tic disorders
TS: Tourette’s syndrome
