# Supplementary material for: Mitochondrial tRNA mutations in Chinese children with tic disorders
Source: Biosci Rep. 2020 Dec 22;40(12):BSR20201856. doi: 10.1042/BSR20201856 (PMC7755120; doi:10.1042/BSR20201856)
Supplement: Supplementary Table S1 [file BSR-2020-1856_supp.pdf]

Supplemental Table S1 The CI was calculated by comparing with 16 Species

Number	Species
1	Bos Taurus
2	Cebus albifrons
3	Gorilla gorilla
4	Homo sapiens
5	Hylobates lar
6	Lemur catta
7	Macaca mulatta
8	Macaca sylvanus
9	Mus musculus
10	Nycticebus coucang
11	Pan paniscus
12	Pan troglodytes
13	Pongo pygmaeus
14	Pongo abelii
15	Papio hamadryas
16	Tarsius bancanus
